# WEALTH LOSS, DEMENTIA, AND COGNITIVE DECLINE: SURFACING NEW CONNECTIONS AND UPSTREAM INTERVENTIONS

**DOI:** 10.1093/geroni/igae098.1271

**Published:** 2024-12-31

**Authors:** Julie Miller, Lucas Yoquinto

**Affiliations:** Chair; Discussant

## Abstract

Nearly one in ten adults in the United States over age 65 has dementia. Another two in ten have mild cognitive impairment: an early, symptomatic period of memory or cognitive decline experienced by those who do not yet meet the full diagnostic criteria for dementia. It has long been a matter of anecdote that serious personal finance losses can coincide with, and arise in the wake of, a dementia diagnosis. This symposium brings together leading researchers to explore financial losses than can occur both before and after formal dementia diagnosis. The first presentation compares household financial trajectories of individuals who ultimately developed dementia with those who did not. The second presentation explores financial status of older adults prior to and following the onset of dementia. The third and final presentation explores changes in marital and health status (including but not limited to transitions from normal cognition to cognitive impairment or dementia) as risk factors for negative wealth shocks in older age. Combined, the presentations surface new insights and questions about connections between financial health and cognitive health. The presentations will be synthesized and brought together by a discussion of possible cross-sector, cross-industry intervention strategies that can be undertaken to protect individuals and families at risk of financial losses associated with dementia, especially in the pre-diagnosis period.

